# Supplementary material for: Unique miRomics Expression Profiles in Tannerella forsythia-Infected Mandibles during Periodontitis Using Machine Learning
Source: Int J Mol Sci. 2023 Nov 16;24(22):16393. doi: 10.3390/ijms242216393 (PMC10671577; doi:10.3390/ijms242216393)
Supplement: Supplementary file 1 [file ijms-24-16393-s001.zip › ijms-2641428-supplementary.pdf]

Unique miRomics Expression Profiles in *Tannerella forsythia*-Infected Mandibles during Periodontitis Using Machine Learning

Chairmandurai Aravindraja [1,†,‡], Syam Jeepipalli [1,†], William Duncan [2], Krishna Mukesh Vekariya [1], Sakshee Bahadekar [3], Edward K. L. Chan [4] and Lakshmyya Kesavalu [1,4,*]

[1] Department of Periodontology, College of Dentistry, University of Florida, Gainesville, FL 32610, USA; aravindrchairman@ufl.edu (C.A.); sjeepipalli@dental.ufl.edu (S.J.); kvekariya@ufl.edu (K.M.V.);
[2] Department of Community Dentistry, College of Dentistry, University of Florida, Gainesville, FL 32610, USA; duncanw@ufl.edu
[3] Department of Computer and Information Science and Engineering, University of Florida, Gainesville, FL 32610, USA; sbahadekar@ufl.edu
[4] Department of Oral Biology, College of Dentistry, University of Florida, Gainesville, FL 32610, USA; echan@dental.ufl.edu
* Correspondence: kesavalu@dental.ufl.edu; Tel.: +1-352-273-6500
† These authors contributed equally to this work.
‡ Current address: Department of Neurology, College of Medicine, University of Florida, Gainesville, FL 32610, USA

Supplementary Data

Table S1. Downsregulated miRNAs, reported functions, and target genes.

**Downregulated miRNAs in 8-weeks *T. forsythia* infection**				
**miRs**	**Fold change**	***p*-value**	**Reported function**	**Number of target genes**
miR-375	-2.38	0.0154379	Down regulated in oral squamous cell carcinoma [1] and salivary adenoid cystic carcinoma [2].	24 *(e.g., Med13, C1qbp, Mtpn, Wdr26, Sept2)*
miR-200c	-2.36	0.01351086	Reduced levels observed in mice infected with LPS of *P. gingivalis* [3].	15 *(e.g., Atp5b, Pls3, Mycn, Zeb1, Thap4)*
miR-200b	-1.86	0.00822482	Variations in the levels of miR-200b observed in gingival tissue of obese periodontitis subjects [4].	100 *(e.g., Ythdf3, N4bp2, Apobec3, G6pc, Sc5d)*
miR-34b-5p	-1.8	0.02779496	Enhance the resistance to bleomycin by regulating its target gene TIMP3 during the pathogenesis of lung fibrosis [5].	14 *(e.g., Mapk1, Mycn, Pou5f1, Sox2, Tfcp2l1)*
miR-141	-1.72	0.01359321	Decreased levels reported in inflamed gingival tissues of periodontitis patients [6].	143 *(e.g., Dlc1, Apob, Acox2, Helz2, Klf5)*
miR-140	-1.48	0.03988881	Downregulated in cancer stem cells of DCIS tumors [7].	
miR-129-5p	-1.45	0.01918338	miR-129-5p down-regulation fosters epithelial to mesenchymal transition in breast cancer [8].	

miR-668	-1.43	0.03277273	Mice infected with lipoteichoic acid has elevated levels which is an opposite observation in our study [9].
miR-205	-1.41	0.03628375	Decreased levels reported in inflamed gingival tissues of periodontitis patients [6].
miR-423-3p	-1.4	0.00303573	Upregulated expression in severe periodontal disease [10]. Reduced expression reported in obese periodontitis subjects [11].
mcmv-miR-m107-1-3p	-1.45	0.0347777	
mmu-miR-883a-5p	-1.44	0.02258418	
mmu-miR-335-5p	-1.44	0.03686075	
mmu-miR-380-5p	-1.43	0.01298791	
mmu-miR-130b	-1.43	0.01389788	
mmu-miR-362-3p	-1.42	0.00463524	
mcmv-miR-m59-2	-1.42	0.01011846	
mmu-miR-290-5p	-1.42	0.02489599	
mmu-miR-484	-1.41	0.01656191	
mmu-miR-1958	-1.41	0.03334294	
mmu-miR-652	-1.4	0.00997111	
mmu-miR-421	-1.4	0.02110001	
mmu-miR-501-5p	-1.4	0.02906832	
mmu-miR-1943	-1.39	0.01257426	
mcmv-miR-m21-1	-1.39	0.02210176	
mghv-miR-M1-2	-1.38	0.03920395	
mcmv-miR-m108-1	-1.38	0.04145297	
mmu-miR-431	-1.37	0.02646171	
mmu-miR-871	-1.37	0.03127912	
mcmv-miR-M55-1	-1.36	0.00405417	
mmu-miR-320	-1.36	0.00729514	
mmu-miR-434-5p	-1.36	0.00919494	
mcmv-miR-m01-1	-1.36	0.01412798	
mcmv-miR-m107-1-5p	-1.36	0.03326055	
mmu-miR-1893	-1.36	0.04138932	
mmu-miR-134	-1.36	0.04317695	
mmu-miR-671-3p	-1.35	0.02214022	
mmu-miR-466d-3p	-1.35	0.02902612	
mmu-miR-105	-1.35	0.04387205	
mmu-miR-1945	-1.35	0.04447647	
mmu-miR-433	-1.34	0.03133953	
mmu-miR-1964	-1.34	0.03661731	
mmu-miR-669i	-1.34	0.04239045	
mmu-miR-374	-1.33	0.01647783	
mmu-miR-1190	-1.33	0.0389211	

mmu-miR-1187	-1.33	0.04878728
mmu-miR-34a	-1.32	0.01648773
mmu-miR-449a	-1.32	0.02795818
mmu-miR-574-5p	-1.32	0.02914963
mmu-miR-370	-1.32	0.03444861
mmu-miR-669e	-1.32	0.03653026
mmu-miR-669j	-1.32	0.0436067
mmu-miR-551b	-1.32	0.04661804
mmu-miR-883b-5p	-1.31	0.02955824
mmu-miR-881	-1.3	0.02161465
mmu-miR-669g	-1.29	0.02050705
mghv-miR-M1-3	-1.29	0.04887573
mmu-miR-2861	-1.28	0.01488577
mmu-miR-1196	-1.28	0.03232638
mmu-miR-384-3p	-1.26	0.02171305
mmu-miR-1306	-1.26	0.04741709
mmu-miR-32	-1.24	0.02447838
mmu-miR-185	-1.24	0.03258776
mmu-miR-497	-1.23	0.03933962
mmu-miR-3473	-1.23	0.04096894
mmu-miR-331-3p	-1.22	0.02712125
mmu-miR-329	-1.21	0.0452145

Details of the target genes were given for the top five significantly expressed miRNAs (miR-375, miR-200c, miR-200b, miR-34b-5p, miR-141) in 8-week infection. Two miRNAs miR-375, miR-200c are highly dysregulated with >2 FC are in blue color. Four miRNAs miR-200b, miR-141, miR-205, miR-423-3p that are dysregulated during 8-week infection (colored in red) are also identified in gingival tissue of periodontitis patients.

Table S2. Downregulated miRNAs, reported functions, and target genes.

**Downregulated miRNAs in 16-weeks *T. forsythia* infection**				
**miRs**	**Fold change**	**p-value**	**Reported functions**	**Number of target genes**
miR-2135	-1.54	5.58E-05	Expressed in upper genital tracts of chlamydia vaccine infection [12].	--
miR-720	-1.45	2.07E-05	Significantly downregulated in primary breast cancer, with greater downregulation in metastatic tumors [13].	--
miR-376c	-1.45	0.00156	Tumor suppressing marker in cholangiocarcinoma cell lines [14].	9 *(e.g., Mycn, Igf2bp1,Gbf1, Mlec, Atf7ip)*
miR-488	-1.38	0.019289	Tumor suppressor molecule in breast cancer [15].	--
miR-322	-1.37	0.000605	Impairing spermatogenesis at decreased expression in GC-2 cells [16].	2761 *(e.g., Arl8b, Syne1, Mul1, Med13, Srrm2)*

miR-150	-1.29	0.024543	Decreased miR-150 levels promoting the cell survival in follicular lymphoma [17]. Serum exosomal miR-150-5p levels were significantly reduced in colorectal cancer cases [18].
miR-335-5p	-1.2	0.034371	Controls the genes related to tumor extracellular matrix function, integrin and Rho signaling [19].
miR-451	-1.18	0.036175	Mice infected with lipoteichoic acid has elevated levels which is an opposite observation in our study [9].
miR-15a	-1.15	0.004286	Downregulated levels observed in Apelin-mediated lung cancer development [20].
miR-342-5p	-1.37	0.002756	
mmu-miR-1937a+mmu-miR-1937b	-1.36	0.001632	
miR-496	-1.36	0.001801	
miR-323-3p	-1.35	0.007892	
miR-1274a	-1.33	0.004807	
miR-130a	-1.33	0.024964	
miR-99a	-1.32	0.007019	
miR-140	-1.3	0.027909	
miR-1937c	-1.29	0.000225	
miR-329	-1.29	0.00581	
miR-362-3p	-1.27	0.034878	
miR-340-5p	-1.25	0.0032	
miR-382	-1.23	0.018191	
miR-126-3p	-1.23	0.043215	
miR-339-5p	-1.21	0.031776	
miR-135a	-1.21	0.035398	
miR-326	-1.19	0.031113	
miR-302b	-1.18	0.012364	
miR-27a	-1.18	0.028277	
miR-292-3p	-1.17	0.033373	
miR-202-3p	-1.17	0.047	
miR-151-3p	-1.16	0.048993	
miR-301a	-1.14	0.017489	

Details of the target genes were given for the top five downregulated significantly expressed miRNAs in 16-week *T. forsythia*-infected mouse mandibles.

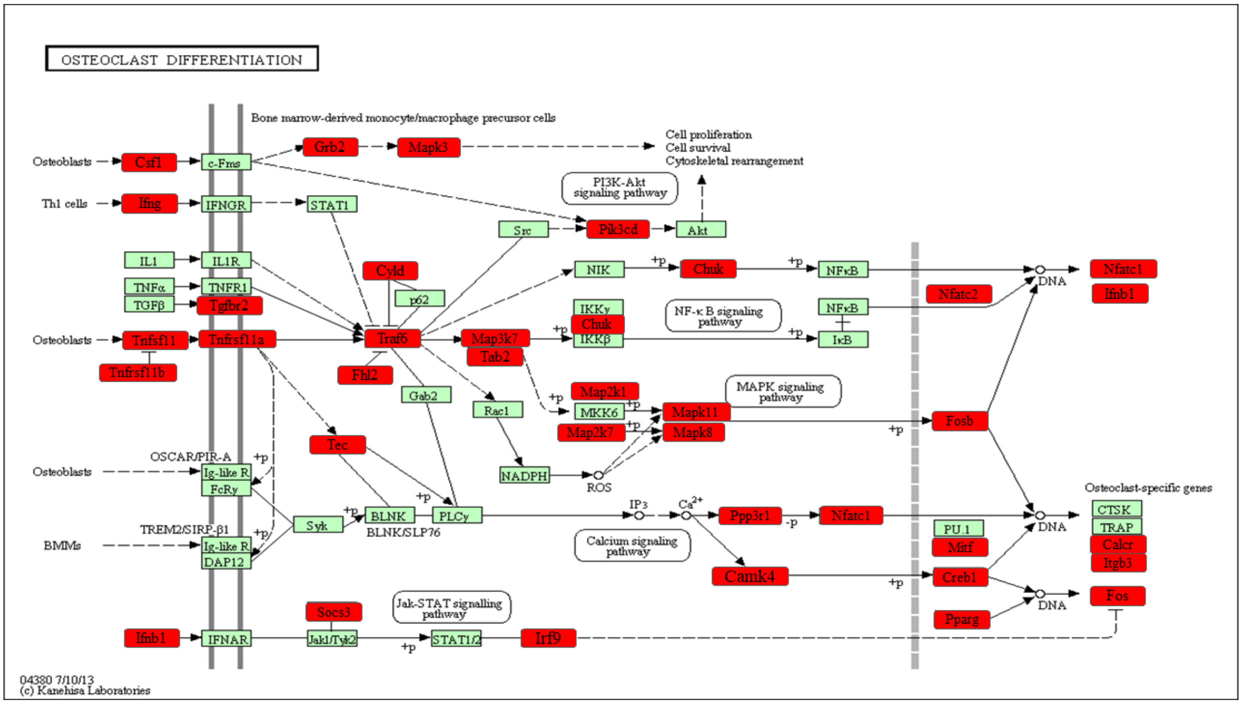

Figure S1. Osteoclast differentiation signaling pathway containing *T. forsythia* significant differentially downregulated genes in mandibles compared with sham-infected controls at $p \leq 0.05$, adapted from pathway express and using the Kyoto Encyclopedia of Genes and Genomes (KEGG). Red boxes indicate significantly decreased 37 genes expression based on downregulated miRNA profiles from Nanostring analysis. Green boxes indicate no change in gene expression. An arrow indicates a molecular interaction resulting in mitogen-activated protein kinase (MAPK) signaling pathway, calcium signaling pathway, Jak-STAT signaling pathway, NF-kB signaling pathway, ubiquitin-mediated proteolysis and a line without an arrowhead indicates a molecular interaction resulting in inhibition. Solid and dash-dotted lines denote direct and indirect relationships, respectively. +P indicates phosphorylation, and −P indicates dephosphorylation. The osteoclasts, multinucleated giant cells originating from the hematopoietic monocyte-macrophage lineage, are responsible for ABR observed in periodontal disease. Osteoclastogenesis is mainly regulated by signaling pathways activated by RANK and immune receptors, whose ligands are expressed on the surface of osteoblasts. Signaling from RANK changes gene expression patterns through transcription factors like NFATc1 and characterizes the active osteoclast.

Table S3. miRTarBase analysis of upregulated DE microRNAs and their target genes in 16 weeks *T. forsythia* infection.

MiRTarBase ID	miRNA	Target Gene
MIRT596881	mmu-miR-1902	*Tsn*
MIRT590085	mmu-miR-1902	*Evi2b*
MIRT594621	mmu-miR-1902	*Myo9a*
MIRT592674	mmu-miR-1902	*Itga11*
MIRT591487	mmu-miR-1902	*Arhgef15*
MIRT747911	mmu-let-7c	*Atp2b2*
MIRT744323	mmu-let-7c	*Ifnar1*
MIRT749593	mmu-let-7c	*Meis2*
MIRT750060	mmu-let-7c	*Nf2*
MIRT743653	mmu-let-7c	*Apc2*
MIRT604532	mmu-miR-423-5p	*Zmat3*
MIRT753281	mmu-miR-210	*Agtrap*
MIRT746151	mmu-miR-210	*Dmd*
MIRT746303	mmu-miR-210	*Lcp2*
MIRT746213	mmu-miR-210	*Foxk1*
MIRT744620	mmu-miR-210	*Ncam1*
MIRT734180	mmu-miR-146a-5p	*PLN*
MIRT734181	mmu-miR-146a-5p	*ANK2*
MIRT054076	mmu-miR-146a-5p	*Relb*
MIRT593074	mmu-miR-146a-5p	*Camk2a*
MIRT579170	mmu-miR-146a-5p	*Cd93*
MIRT743884	mmu-miR-99b	*Comp*
MIRT750116	mmu-miR-99b	*Grik3*
MIRT745042	mmu-miR-99b	*Slc35d2*
MIRT746080	mmu-miR-99b	*Asxl2*
MIRT599336	mmu-miR-99b-5p	*Comp*
MIRT747906	mmu-let-7a	*Atp2b2*
MIRT744330	mmu-let-7a	*Ifnar1*
MIRT749595	mmu-let-7a	*Meis2*
MIRT750058	mmu-let-7a	*Nf2*
MIRT743651	mmu-let-7a	*Apc2*
MIRT744237	mmu-miR-127	*Gpi1*
MIRT747400	mmu-miR-127	*Tspan13*
MIRT744805	mmu-miR-127	*Polr3k*
MIRT745089	mmu-miR-127	*Spc24*
MIRT744830	mmu-miR-127	*Prkrir*

MIRT747908	mmu-miR-98	*Atp2b2*
MIRT744324	mmu-miR-98	*Ifnar1*
MIRT749591	mmu-miR-98	*Meis2*
MIRT750053	mmu-miR-98	*Nf2*
MIRT743648	mmu-miR-98	*Apc2*
MIRT745638	mmu-miR-24	*Chrna1*
MIRT745611	mmu-miR-24	*Birc5*
MIRT745704	mmu-miR-24	*Eif4g2*
MIRT743683	mmu-miR-24	*B4galnt2*
MIRT744163	mmu-miR-24	*Ggps1*
MIRT749453	mmu-miR-876-3p	*1700054N08Rik*
MIRT586837	mmu-miR-876-3p	*Il18r1*
MIRT601907	mmu-miR-876-3p	*Padi2*
MIRT597801	mmu-miR-876-3p	*Pla2r1*
MIRT605365	mmu-miR-876-3p	*Vldlr*
MIRT748528	mmu-miR-218	*Prdm1*
MIRT752329	mmu-miR-218	*Bsn*
MIRT750688	mmu-miR-218	*Eno2*
MIRT750206	mmu-miR-218	*Nab1*
MIRT749367	mmu-miR-218	*Sh2d1a*
MIRT750305	mmu-miR-218	*Sim1*
MIRT752327	mmu-miR-101b	*Bsn*
MIRT749944	mmu-miR-101b	*Cebpa*
MIRT751505	mmu-miR-101b	*Chml*
MIRT752707	mmu-miR-101b	*Mapre1*
MIRT750799	mmu-miR-101b	*Hif1a*
MIRT753366	mmu-miR-23b	*Il17ra*
MIRT753387	mmu-miR-23b	*Magea5*
MIRT744530	mmu-miR-23b	*Mbd4*
MIRT744688	mmu-miR-23b	*Nrp2*
MIRT747241	mmu-miR-23b	*Ptpre*
MIRT747910	mmu-let-7e	*Atp2b2*
MIRT744322	mmu-let-7e	*Ifnar1*
MIRT749590	mmu-let-7e	*Meis2*
MIRT750055	mmu-let-7e	*Nf2*
MIRT743647	mmu-let-7e	*Apc2*
MIRT745057	mmu-miR-26b	*Slc7a1*
MIRT749019	mmu-miR-26b	*Kpna2*
MIRT749425	mmu-miR-26b	*Pten*

MIRT752818	mmu-miR-26b	*Nus1*
MIRT752922	mmu-miR-26b	*Rgs17*

We used mmu-miR-1902 an example for an upregulated DE miRNA during 16 weeks of infection in identifying the target genes using the miRTarBase. Each miRNA has different target genes and each with a specific MiRTarBase ID. *T. forsythia*-infection induced DE upregulated mmu-miR-1902 has 5 different target genes with 5 different MiRTarBase ID as stated in the table. Similarly, to each miRNA, the first five MiRTarBase IDs and # of target genes shown in the table. The details of other 15 upregulated miRNAs and their # of target genes and specific MiRTarBase IDs in brackets as follows, mmu-let-7c (37), mmu-miR-423-5p (1), mmu-miR-210 (45), mmu-miR-146a (29), mmu-miR-99b (8), mmu-let-7a (45), mmu-miR-127 (19), mmu-miR-98 (35), mmu-miR-24 (438), mmu-miR-876-3p (19), mmu-miR-218 (38), mmu-miR-101b (44), mmu-miR-23b (50), mmu-let-7e (39) and mmu-miR-26b (38).

References

1. Siow, M.Y.; Ng, L.P.; Vincent-Chong, V.K.; Jamaludin, M.; Abraham, M.T.; Abdul Rahman, Z.A.; Kallarakkal, T.G.; Yang, Y.H.; Cheong, S.C.; Zain, R.B. Dysregulation of miR-31 and miR-375 expression is associated with clinical outcomes in oral carcinoma. *Oral Dis* **2014**, *20*, 345-351, doi:10.1111/odi.12118.
2. Mitani, Y.; Roberts, D.B.; Fatani, H.; Weber, R.S.; Kies, M.S.; Lippman, S.M.; El-Naggar, A.K. MicroRNA profiling of salivary adenoid cystic carcinoma: association of miR-17-92 upregulation with poor outcome. *PLoS One* **2013**, *8*, e66778, doi:10.1371/journal.pone.0066778.
3. Krongbaramee, T.; Zhu, M.; Qian, Q.; Zhang, Z.; Eliason, S.; Shu, Y.; Qian, F.; Akkouch, A.; Su, D.; Amendt, B.A.; et al. Plasmid encoding microRNA-200c ameliorates periodontitis and systemic inflammation in obese mice. *Mol Ther Nucleic Acids* **2021**, *23*, 1204-1216, doi:10.1016/j.omtn.2021.01.030.
4. Kalea, A.Z.; Hoteit, R.; Suvan, J.; Lovering, R.C.; Palmen, J.; Cooper, J.A.; Khodiyar, V.K.; Harrington, Z.; Humphries, S.E.; D'Aiuto, F. Upregulation of gingival tissue miR-200b in obese periodontitis subjects. *J Dent Res* **2015**, *94*, 59S-69S, doi:10.1177/0022034514568197.
5. Hu, R.P.; Lu, Y.Y.; Zhang, X.J. MiR-34b-5p knockdown attenuates bleomycin-induced pulmonary fibrosis by targeting tissue inhibitor of metalloproteinase 3 (TIMP3). *Eur Rev Med Pharmacol Sci* **2019**, *23*, 2273-2279, doi:10.26355/eurrev_201903_17276.
6. Stoecklin-Wasmer, C.; Guarnieri, P.; Celenti, R.; Demmer, R.T.; Kebschull, M.; Papapanou, P.N. MicroRNAs and their target genes in gingival tissues. *J Dent Res* **2012**, *91*, 934-940, doi:10.1177/0022034512456551.
7. Li, Q.; Yao, Y.; Eades, G.; Liu, Z.; Zhang, Y.; Zhou, Q. Downregulation of miR-140 promotes cancer stem cell formation in basal-like early stage breast cancer. *Oncogene* **2014**, *33*, 2589-2600, doi:10.1038/onc.2013.226.
8. Yu, Y.; Zhao, Y.; Sun, X.H.; Ge, J.; Zhang, B.; Wang, X.; Cao, X.C. Down-regulation of miR-129-5p via the Twist1-Snail feedback loop stimulates the epithelial-mesenchymal transition and is associated with poor prognosis in breast cancer. *Oncotarget* **2015**, *6*, 34423-34436, doi:10.18632/oncotarget.5406.
9. Hsieh, C.H.; Yang, J.C.; Jeng, J.C.; Chen, Y.C.; Lu, T.H.; Tzeng, S.L.; Wu, Y.C.; Wu, C.J.; Rau, C.S. Circulating microRNA signatures in mice exposed to lipoteichoic acid. *J Biomed Sci* **2013**, *20*, 2, doi:10.1186/1423-0127-20-2.
10. Costantini, E.; Sinjari, B.; Di Giovanni, P.; Aielli, L.; Caputi, S.; Muraro, R.; Murmura, G.; Reale, M. TNFalpha, IL-6, miR-103a-3p, miR-423-5p, miR-23a-3p, miR-15a-5p and miR-223-3p in the crevicular fluid of periodontopathic patients correlate with each other and at different stages of the disease. *Sci Rep* **2023**, *13*, 126, doi:10.1038/s41598-022-26421-6.
11. Naqvi, A.R.; Brambila, M.F.; Martinez, G.; Chapa, G.; Nares, S. Dysregulation of human miRNAs and increased prevalence of HHV miRNAs in obese periodontitis subjects. *J Clin Periodontol* **2019**, *46*, 51-61, doi:10.1111/jcpe.13040.
12. Howard, S.; Richardson, S.; Benyeogor, I.; Omosun, Y.; Dye, K.; Medhavi, F.; Lundy, S.; Adebayo, O.; Igietseme, J.U.; Eko, F.O. Differential miRNA Profiles Correlate With Disparate Immunity Outcomes Associated With Vaccine Immunization and Chlamydial Infection. *Front Immunol* **2021**, *12*, 625318, doi:10.3389/fimmu.2021.625318.
13. Li, L.Z.; Zhang, C.Z.; Liu, L.L.; Yi, C.; Lu, S.X.; Zhou, X.; Zhang, Z.J.; Peng, Y.H.; Yang, Y.Z.; Yun, J.P. miR-720 inhibits tumor invasion and migration in breast cancer by targeting TWIST1. *Carcinogenesis* **2014**, *35*, 469-478, doi:10.1093/carcin/bgt330.

14. Iwaki, J.; Kikuchi, K.; Mizuguchi, Y.; Kawahigashi, Y.; Yoshida, H.; Uchida, E.; Takizawa, T. MiR-376c down-regulation accelerates EGF-dependent migration by targeting GRB2 in the HuCCT1 human intrahepatic cholangiocarcinoma cell line. *PLoS One* **2013**, *8*, e69496, doi:10.1371/journal.pone.0069496.
15. Wu, Y.; Yuan, M.H.; Wu, H.T.; Chen, W.J.; Zhang, M.L.; Ye, Q.Q.; Liu, J.; Zhang, G.J. MicroRNA-488 inhibits proliferation and motility of tumor cells via downregulating FSCN1, modulated by Notch3 in breast carcinomas. *Cell Death Dis* **2020**, *11*, 912, doi:10.1038/s41419-020-03121-5.
16. Che, Q.; Wang, W.; Duan, P.; Fang, F.; Liu, C.; Zhou, T.; Li, H.; Xiong, C.; Zhao, K. Downregulation of miR-322 promotes apoptosis of GC-2 cell by targeting Ddx3x. *Reprod Biol Endocrinol* **2019**, *17*, 63, doi:10.1186/s12958-019-0506-7.
17. Musilova, K.; Devan, J.; Cerna, K.; Seda, V.; Pavlasova, G.; Sharma, S.; Oppelt, J.; Pytlik, R.; Prochazka, V.; Prouzova, Z.; et al. miR-150 downregulation contributes to the high-grade transformation of follicular lymphoma by upregulating FOXP1 levels. *Blood* **2018**, *132*, 2389-2400, doi:10.1182/blood-2018-06-855502.
18. Zou, S.L.; Chen, Y.L.; Ge, Z.Z.; Qu, Y.Y.; Cao, Y.; Kang, Z.X. Downregulation of serum exosomal miR-150-5p is associated with poor prognosis in patients with colorectal cancer. *Cancer Biomark* **2019**, *26*, 69-77, doi:10.3233/CBM-190156.
19. Winkler, I.; Bitter, C.; Winkler, S.; Weichenhan, D.; Thavamani, A.; Hengstler, J.G.; Borkham-Kamphorst, E.; Kohlbacher, O.; Plass, C.; Geffers, R.; et al. Identification of Ppargamma-modulated miRNA hubs that target the fibrotic tumor microenvironment. *Proc Natl Acad Sci U S A* **2020**, *117*, 454-463, doi:10.1073/pnas.1909145117.
20. Ran, J.; Li, Y.; Liu, L.; Zhu, Y.; Ni, Y.; Huang, H.; Liu, Z.; Miao, Z.; Zhang, L. Apelin enhances biological functions in lung cancer A549 cells by downregulating exosomal miR-15a-5p. *Carcinogenesis* **2021**, *42*, 243-253, doi:10.1093/carcin/bgaa089.
